# Attention-deficit/hyperactivity symptoms in preschool children from an E-waste recycling town: assessment by the parent report derived from DSM-IV

**DOI:** 10.1186/s12887-015-0368-x

**Published:** 2015-05-05

**Authors:** Ruibiao Zhang, Xia Huo, Guyu Ho, Xiaojuan Chen, Hongwu Wang, Tianyou Wang, Lian Ma

**Affiliations:** 1 Department of Pediatrics, Second Affiliated Hospital of Shantou University Medical College, Shantou, Guangdong People’s Republic of China; 2 Analytical Cytology Laboratory and Guangdong Provincial Key Laboratory of Infectious Diseases and Molecular Immunopathology, Shantou University Medical College, Shantou, Guangdong People’s Republic of China; 3 Translational Medicine Center, Shantou University Medical College, Shantou, Guangdong People’s Republic of China; 4 Department of Hematology Oncology Center, Beijing Children’s Hospital, Capital Medical University, Beijing, People’s Republic of China; 5 Shenzhen Pingshan Maternal and Child Health Hospital, Shenzhen, Guangdong People’s Republic of China; 6 Shenzhen University Women’s and Children’s Hospital, Shenzhen, Guangdong People’s Republic of China

**Keywords:** Behavioral disorder, ADHD, Child, Electronic waste, Lead, Cadmium

## Abstract

**Background:**

To investigate the attention-deficit/hyperactivity disorder (ADHD) status among preschool-aged children in Guiyu, an electronic waste (e-waste) recycling town in Guangdong, China.

**Methods:**

Two hundred and forty-three parents were surveyed regarding ADHD behaviors in their children (aged 3–7 years) based solely on the DSM-IV criteria. The peripheral blood samples were taken from these children to measure blood lead levels (BLLs) and blood cadmium levels (BCLs).

**Results:**

12.8% of children met the criteria for ADHD, of which the inattentive, hyperactive/impulsive and combined subtypes were 4.5%, 5.3% and 2.9% respectively. Of all children, 28.0% had BLLs ≥ 10 ug/dL and only 1.2% had BCLs ≥ 2 ug/L, levels conventionally considered high. Either modeled by univariate or multivariable analysis, the three ADHD scores (inattentive, hyperactive/impulsive and total scores) calculated from the Parent Rating Scale showed strong positive correlations with BLLs but not with BCLs. Furthermore, children with high BLLs had 2.4 times higher risk of ADHD than those with low BLLs (OR: 2.4 [95% CI: 1.1–5.2]). When each of the 18 categories on the Parent Rating Scale was separately analyzed, children with high BLLs had significant higher risks for positive ADHD symptoms than those with low BLLs in 12 of the 18 categories (ORs ranged from 2.1 [95% CI: 1.1–3.9] to 3.6 [95% CI: 1.7–7.5]).

**Conclusions:**

This study suggests that environmental lead contamination due to e-waste recycling has an impact on neurobehavioral development of preschool children in Guiyu.

**Electronic supplementary material:**

The online version of this article (doi:10.1186/s12887-015-0368-x) contains supplementary material, which is available to authorized users.

## Background

Electronic waste (e-waste) is the most rapidly growing waste problem in the world. But to date, consumers, industry and government have only taken small steps to deal with this looming problem. Furthermore, some developed countries such as United States, Canada, Japan, and European countries that generate overwhelming majority of the hazardous waste have made use of exporting the e-waste crisis to the developing countries of Asia [1,2].

Due to its geographical location, Guiyu, a seaside town situated in the southern coast of China, is one of the largest e-waste destinations in the world. It has a total area of 52 km^2^ with a population of 133,000 (in 2008). Nearly 60–80% of families in the town are engaged in e-waste recycling operations. The hazardous recycling methods are mainly as follows: sorting, firing, incinerating, acidic/alkaline bathing, manual disassembling, open burning of wires and cables and strong acid leaching [3]. By such primitive means, approximately 1.7 million tons of e-waste are dismantled annually, threatening the local environment and resident health. Several studies have reported that the Guiyu environment has soaring levels of toxic heavy metals and organic contaminants in workplace environment, surrounding soil and water sources [4-7] and that people engaging in e-waste recycling operations in Guiyu have high incidence of skin damage, headaches, vertigo, nausea, chronic gastritis, gastric and duodenal ulcers [8]. Lead (Pb) and cadmium (Cd) are widely used in electronic devices and our previous studies reported that Guiyu children had significantly higher blood lead levels (BLLs) and blood cadmium levels (BCLs) than those from Chendian (a neighboring town) and other suburban areas [9]. Thus, the two potential risk factors were chosen to study their associations with attention-deficit/hyperactivity disorder (ADHD).

ADHD is the most common childhood neurobehavioral abnormalities, characterized by inattention, impulsivity and hyperactivity. Because of the lack of laboratory tests that can reliably predict ADHD, the diagnosis depends heavily on the interviews and questionnaires [10]. In the epidemiologic research, the rating scales derived from 1994 Diagnostic and Statistical Manual of Mental Disorders (DSM-IV) criteria are widely used and also recommended by the American Academy of Pediactrics (AAP) for the ADHD evaluation in 2000 [11]. In addition, most epidemiologic publications we searched regarding ADHD used the DSM-IV Rating Scale [12-14]. We therefore chose the same rating system to assess the prevalence of ADHD in this study.

The ADHD heritability, estimated at 60% to 80%, highlights the considerable role of environmental toxicants in the disorder susceptibility [15]. It is likely that Guiyu children are at great risk of ADHD, because e-waste contains hundreds of toxicants and some of them such as lead, cadmium, manganese, and polycyclic aromatic hydrocarbons (PAHs) have been demonstrated to be associated with ADHD [16-19]. Moreover, unknown interactions among these toxicants may have compounding effects on the development of ADHD and thus Guiyu children could be more susceptible. Unfortunately, there are very few investigations regarding ADHD in this special environment. Therefore, we conducted the study to evaluate the effect of e-waste pollution on children with ADHD in Guiyu.

## Methods

### Study participants and sample collection

This cross-sectional study carried out during January 2012 to May 2012 in Guiyu, China (a latitude and longitude of 23.29–23.41°N and 116.30–116.40°E). We chose 243 children aged 3–7 years from two public kindergartens as study participants. All children were born in Guiyu and were not diagnosed as ADHD before. Unhealthy children or those on medications were excluded. The study was approved by the Human Ethics Committee of Shantou University Medical College and all participating parents gave their written informed consents (about 25% parents refused to participate in the study). A self-designed questionnaire was used to conduct the survey among parents (see Additional file 1). The questionnaire included factors that might impact on children’s ADHD behaviors including nutrition intake, residence, household tobacco smoke exposure, father’s work relating to e-waste, parents’ education levels and monthly household income (Poor: less than 2000 yuan; Moderate: 2000–5000 yuan; Good: more than 5000 yuan), and so forth.

Blood samples (3 ml) were collected by well-trained nurses, transported to the laboratory, and stored at –20°C until analysis. One part of the blood sample (1 ml) was used to measure levels of Pb and Cd. The other part (2 ml) was used to measure serum ferritin by the chemiluminescence method (Advia Centaur XP, Semins, German) for the preliminary assessment of the nutritional status.

### Assessment of ADHD

For assessing ADHD, a Chinese-version of the Parent Rating Scale adapted from the DSM-IV criteria was used. On the ADHD Parent Rating Scale, there are 18 categories related to ADHD behavioral symptoms (see Additional file 1). The parents were asked to rate their children on each of the categories on the scale of 0 (never or rarely), 1 (sometimes), 2 (often) and 3 (very often). The scores from each category were added to form three scores: inattentive score (sum of items 1 to 9), hyperactive/impulsive score (sum of items 10 to 18) and total score (sum of items 1 to 18). Each category with a score of 2 or 3 was considered as a single positive ADHD symptom. Three subtypes of ADHD based on the DSM-IV criteria were as follows: inattentive type, at least 6 positive ADHD symptoms on the inattention subscale (category 1 to 9) but not on the hyperactivity/impulsivity subscale (category 9 to 18); hyperactive/impulsive type, at least 6 positive ADHD symptoms on the hyperactivity/impulsivity subscale but not on the inattention subscale; combined type, met criteria for both inattentive and hyperactive/impulsive types.

### Measurement of blood Pb and Cd

Before analyses, 100 μL blood samples were added to 900 μl of 0.5% nitric acid (for blood cadmium, 2% nitric acid), vortexed, digested at room temperature for 10 min, and the digest was then used for Pb determination. The mixture was then used for the lead determination. The supernatant of the digest was obtained by centrifuging for 10 min at 2,000 rpm and used for Cd analysis.

Pb and Cd were determined by the graphite furnace atomic absorption spectrophotometry (Jena Zenit 650, Germany), which consists of an autosampler (MPE60) with an injection volume set at 20 μL. The main parameters used for the lead determination were: a wave length of 283.3 nm, a lamp current of 4.0 mA, a slit width of 0.8 nm, drying at 90°C, 105°C and 120°C, ashing at 600°C, and atomization at 1,500°C. The standard calibration curve was plotted using the six working standard solutions which were prepared from the stock Pb standard solution diluted with nitric acid and added matrix modifier mixed with human blood. The linear correlation coefficient of the Pb standard calibration curve was 0.9920. Accuracy of the method was controlled by recoveries between 96% and 108%. The parameters for Cd analysis were: a wave length of 228.8 nm, a current of 2.0 mA, a slit width of 1.2 nm, drying at 90°C, 105°C and 120°C, ashing at 300°C, and atomization at 1,300°C. The linear correlation coefficient of the Cd standard calibration curve was 0.9990. The recoveries for this method were 100–103%, which were also from spiked blood samples.

### Statistical analyses

All database management and statistical analyses were conducted with SPSS 13.0 software (SPSS, Inc., Chicago, IL, USA). The central tendency of data was expressed as mean ± SD or median (5th to 95th percentile interval). The Spearman rank correlation (r_s_) was used to assess any univariate associations. The Kormogorov-Smirnov test was used to determine the distribution of each set of data. Because the distribution of ADHD scores was skewed, log-transform of ADHD scores was used in linear regression analysis. A Chi-square test was used to compare categorical variables between different groups. Two-tailed p values < 0.05 were considered statistically significant.

## Results

Parameters of the study population regarding age, sex, serum ferritin, socioeconomic characteristics of the family, residence, household tobacco smoke exposure, blood Pb and Cd, and ADHD scores are presented in Table 1. 28% (68/243) of children tested had BLLs ≥ 10 ug/dL, which is considered high as defined by the US Center for Disease Control [20]. For the threshold value of blood Cd, 5 ug/L was reported as a risk for intoxication [21]. Because all children tested had BCLs < 5 ug/L, we used 2 ug/L as the cut-off point of BCLs empirically. Nevertheless, only 1.2% (3/243) of children had BCLs ≥ 2 ug/L. 12.8% (31/243) of children tested met the DSM-IV criteria for ADHD by the Parent Rating Scale. The ADHD subtypes of inattensive, hyperactive/impulsive and combined types were 4.5% (11/243), 5.3% (13/243) and 2.9% (7/243), respectively.

**Table 1:** **Participant characteristics and data on e-waste exposure and ADHD among 243 preschool children in Guiyu**

Characteristics	Values
Age (years)	5.1 ± 1.0
Male, n (%)	141 (58.0%)
Serum ferritin (ug/L)	51.8 (20.3–113.9)
Father’s education level, n (%)	
Illiterate/primary school	26 (11.1%)
Middle school	145 (61.7%)
High school	52 (22.1%)
College	12 (5.1%)
Mother’s education level, n (%)	
Illiterate/primary school	45 (19.3%)
Middle school	134 (57.5%)
High school	38 (16.3%)
College	16 (6.9%)
Monthly household income, n (%)	
Poor, n (%)	38 (16.5%)
Moderate, n (%)	135 (58.4%)
Good, n (%)	58 (25.1%)
Father’ s work relating to e-waste, n (%)	
Yes	98 (42.8%)
No	131 (57.2%)
E-waste workshops around the house, n (%)	
Yes	137 (58.3%)
No	98 (41.7%)
Household tobacco smoke exposure, n (%)	
Yes	170 (70.8%)
No	70 (29.2%)
**Status of e-waste exposure**	
Blood Pb (ug/dL)	7.9 (5.1–16.9)
Blood Cd (ug/L)	0.95 (0.54–1.57)
Blood Pb ≥ 10 ug/dL, n (%)	68 (28.0%)
Blood Cd ≥ 2 ug/L, n (%)	3 (1.2%)
**Status of DSM-IV ADHD**	
Inattentive score	7.9 ± 4.7
Hyperactive/Impulsive score	7.2 ± 4.9
Total score^a^	15.2 ± 8.6
Inattentive type, n (%)	11 (4.5%)
Hyperactive/Impulsive type, n (%)	13 (5.3%)
Combined type, n (%)^b^	7 (2.9%)
ADHD, n (%)^c^	31 (12.8%)

Values are arithmetic mean ± SD, median (5th to 95th percentile interval) and percentage.

^a^Total score: sum of inattentive and hyperactive/impulsive scores.

^b^Combined type: met criteria for both inattentive and hyperactive/impulsive types.

^c^ADHD: sum of inattentive, hyperactive/impulsive and combined types.

In the initial evaluation, we assessed the univariate association between ADHD scores and various ADHD-related variables (Table 2). Father’s work relating to e-waste, e-waste workshops around the house, household tobacco smoke exposure and BCLs contributed to the high BLLs. The three ADHD scores (inattentive, hyperactive/impulsive and total scores) calculated from the Parent Rating Scale had positive correlations with BLLs, father’s work in e-waste processing and e-waste workshops around the house, negative correlations with sex, age and serum ferritin, but no correlation with BCLs. We further evaluated the association of ADHD scores with BLLs by adjusting for the above 5 statistically significant factors (Table 3). In this multiple regression analysis, BLLs were shown as a major contributing factor to the increased ADHD scores (all three scores).

**Table 2:** **Spearman correlation coefficients (r**_**s**_ **) among ADHD scores and ADHD-related variables**

N = 243	BLLs	BCLs	IA score ^ a ^	HI score ^ b ^	Total score ^ c ^
Age	–0.118	0.066	–0.259**	–0.155*	–0.227**
Sex	–0.196**	0.021	–0.154*	–0.263**	–0.246**
Ferritin	–0.069	–0.055	–0.137*	–0.148*	–0.158*
BLLs	--	0.141*	0.246**	0.317**	0.309**
BCLs	0.141*	--	–0.030	0.024	0.015
Inattentive score	0.246**	–0.030	--	0.581**	0.883**
Hyperactive/Impulsive score	0.317**	0.024	0.581**	--	0.879**
Total score	0.309**	0.015	0.883**	0.879**	--
Father’s education level	–0.247**	–0.025	0.012	0.041	0.019
Mother’s education level	–0.206**	0.048	–0.114	–0.084	–0.115
Monthly household income	–0.182**	–0.019	–0.075	–0.034	–0.060
Father’s work relating to e-waste	0.202**	0.113	0.177**	0.106	0.145*
E-waste workshops around the house	0.147*	0.142*	0.232**	0.169**	0.219**
Household tobacco smoke exposure	0.138*	–0.102	0.090	0.069	0.081

*p < 0.05; **p < 0.01.

^a^IA score: inattentive score.

^b^HI score: hyperactive/impulsive score.

^c^Total score: sum of inattentive and hyperactive/impulsive scores.

**Table 3:** **Multiple linear regression analyses between ADHD scores and BLLs after adjusting for ADHD-related variables**

Dependent variables ^ a ^	Independent variables	Beta ^ b ^	p
**Model 1**	R^2^	0.18	
Inattentive score	Blood lead levels	0.225	0.001
	Age	–0.210	0.001
	Sex	–0.098	0.119
	Serum ferritin	–0.005	0.940
	Father’s work relating to e-waste	0.010	0.879
	E-waste workshops around the house	0.171	0.011
**Model 2**	R^2^	0.20	
Hyperactive/Impulsive score	Blood lead levels	0.283	0.000
	Age	–0.148	0.020
	Sex	–0.176	0.005
	Serum ferritin	–0.035	0.579
	Father’s work relating to e-waste	–0.046	0.490
	E-waste workshops around the house	0.150	0.024
**Model 3**	R^2^	0.22	
Total score^c^	Blood lead levels	0.284	0.000
	Age	–0.198	0.002
	Sex	–0.154	0.012
	Serum ferritin	–0.022	0.715
	Father’s work relating to e-waste	–0.021	0.751
	E-waste workshops around the house	0.179	0.006

^a^Log-transformed values.

^b^Standardized coefficients.

^c^Total score: sum of inattentive and hyperactive/impulsive scores.

When separating into high and low BLLs groups, we found that children with high BLLs had 2.4 times higher risk of ADHD than those with low BLLs (OR: 2.4 [95% CI: 1.1–5.2]) (Table 4). When each of the 18 categories on the Parent Rating Scale was separately analyzed, children with high BLLs showed significant higher rates of positive ADHD symptoms than those with low BLLs in 12 of the 18 categories (ORs ranged from 2.1 [95% CI: 1.1–3.9] to 3.6 [95% CI: 1.7–7.5]) (Table 5).

**Table 4:** **Comparison of the rates of total individuals diagnosed as ADHD between high (N = 68) and low BLLs (N = 175) groups**

	BLLs ≥ 10 ug/dL	BLLs < 10 ug/dL	*χ* ^ 2 ^	p	OR (95% CI)
	n %	n %			
With ADHD^a^	14 20.5	17 9.7	5.203	0.023	2.4 (1.1–5.2)

^a^Total individuals that met the criteria of inattentive, hyperactive/impulsive and combined types.

CI, Confidence interval.

**Table 5:** **Comparison of the positive ADHD symptom rates between high (N = 68) and low BLLs (N = 175) groups**

Items	BLLs ≥ 10 ug/dL % (n)	BLLs < 10 ug/dL % (n)	*χ* ^ 2 ^	p	OR (95% CI)
**Inattention subscale**					
1. Fails to pay attention	30.9 (21)	22.3 (39)	1.946	0.163	1.6 (0.8–2.9)
2. Difficult to sustain attention	29.4 (20)	14.3 (25)	7.426	0.006	2.5 (1.3–4.9)
3. Does not seem to listen	23.5 (16)	14.3 (25)	2.983	0.084	1.8 (0.9–3.7)
4. Does not follow through	14.7 (10)	8.6 (15)	1.997	0.158	1.8 (0.8–4.3)
5. Difficulty organizing	13.2 (9)	6.9 (12)	2.523	0.112	2.1 (0.8–5.2)
6. Avoids mental effort	23.5 (16)	12.6 (22)	4.457	0.035	2.1 (1.1–4.4)
7. Loses things	35.3 (24)	17.1 (30)	9.335	0.002	2.6 (1.4–5.0)
8. Easily distracted	42.6 (29)	21.7 (38)	10.745	0.001	2.7 (1.5–4.9)
9. Forgetful	23.5 (16)	12.0 (21)	5.043	0.025	2.3 (1.1–4.6)
**Hyperactivity/Impulsivity subscale**					
10. Fidgets	52.9 (36)	25.7 (45)	16.336	0.000	3.3 (1.8–5.8)
11. Leaves seat	20.6 (14)	10.3 (18)	4.546	0.033	2.3 (1.1–4.9)
12. Runs about	26.5 (18)	9.1 (16)	12.218	0.000	3.6 (1.7–7.5)
13. Has difficulty playing	19.1 (13)	9.1 (16)	4.636	0.031	2.3 (1.1–5.2)
14. Is “on the go”	36.8 (25)	21.7 (38)	5.776	0.016	2.1 (1.1–3.9)
15. Talks excessively	35.3 (24)	18.3 (32)	7.988	0.005	2.4 (1.3–4.6)
16. Blurts out answer	13.2 (9)	6.3 (11)	3.131	0.077	2.3 (0.9–5.8)
17. Difficulty waiting turn	17.6 (12)	5.7 (10)	8.469	0.004	3.5 (1.4–8.6)
18. Interrupts or intrudes	19.1 (13)	12.6 (22)	1.702	0.192	1.6 (0.8–3.5)

CI, Confidence interval.

## Discussion

Our study showed that the prevalence of ADHD in the e-waste recycling town Guiyu and that children with high BLLs were associated with an increased risk of ADHD. The study would facilitate a better policy making to safe-guard the environment and children living in the area.

The prevalence of ADHD is generally accepted to be around 3–5% among school-aged children [22]. ADHD is most likely a developmental disorder and the onset of ADHD behaviors is usually noted during the preschool years. More frequent or salient ADHD behaviors might be seen in younger children, which gradually decline as children mature [10]. In this study, we found that the preschool children had a prevalence rate of ADHD at 12.8%, which was markedly higher than 5%. However, comparing the rate of ADHD with other regions in preschool-aged children is complicated due to different methods used in selecting study populations, demographic characteristics, ethnicity and culture [23]. Due to the use of DSM-IV ADHD Parent Rating Scale, some publications might provide us with a compatible baseline. Gimpel GA et al. reported that 9.5% of children aged 2–6 years in the US were diagnosed as ADHD in a study population of 253 children [24]. Other researchers in Iceland and Greece reported the prevalence rates were 4.7% and 6.5%, respectively [25,26]. The higher incidence rate of ADHD in Guiyu suggests that the primitive e-waste recycling operations may have an impact on neurobehavioral development of preschool children.

As shown in this study, the three ADHD scores (inattentive, hyperactive/impulsive and total scores) were associated with evaluated BLLs in the univariate analysis. This is consistent with numerous publications [27,28]. However, if we wanted to analyze the relationship between e-waste exposure and ADHD, considering a single lead exposure may not suffice because the e-waste exposure contains lots of toxicants such as mercury, manganese and PAHs. Due to the limitation of expense and test blood volume, we opted to investigate the children’s living conditions instead of measuring other toxicant levels. In our study, the father’s work relating to e-waste and e-waste workshops around the house were both associated with ADHD scores, which might reflect that the primitive e-waste recycling activities have contributed to the cause of childhood ADHD. It is possible that parents engaging in e-waste recycling work could carry e-waste contaminates home and children living around e-waste workshops could intake e-waste toxicants with more outdoor activities [29]. Other factors such as sex, age, serum ferritin, family income and household tobacco smoke exposure should be considered in the evaluation of ADHD. Researchers reported that boys identified with ADHD were at least 4 times higher than girls and the age was significantly associated with the decline in ADHD symptoms [30,31]. Some authors have noted that low serum ferritin levels were correlated with more ADHD symptoms as measured by Conners’ Parent Rating Scales (CPRS) [32]. Research conducted among 493 White and African American children from birth through age 5 indicated that family income affected maternal emotional distress, which indirectly impacted on children’s behaviors [33]. In addition, children with reported secondhand smoke exposure at home had 1.5 times higher risk of ADHD than those who were not exposed [34].

When we put the above ADHD-related factors together by multiple linear regression analyses, we found that the high BLLs still played a major role in the development of ADHD. Furthermore, we found that the children with high BLLs had 2.4 times higher risk of ADHD than the children with low BLLs. The observation is supported by a recent analysis from the 2001–2004 National Health and Nutrition Examination Survey [35]. It showed that children with higher BLLs (1.3–5 ug/dL) had a more than twofold increased risk of ADHD as compared with those with lower BLLs (non-detectable to 0.8 ug/dL).

Cadmium is another heavy metal that has received considerable concern about the profound effect on children’s behavior and neurological development. It has been reported that cadmium levels in children are positively correlated with learning difficulties and dyslexia [36]. Study conducted among 149 children aged 5–16 years showed that the presence of cadmium considerably affected the measurements of their intelligence, physical fitness and academic achievement [37]. A recent report also revealed a higher frequency of withdrawal, social problems, and attention problems associated with higher levels of cadmium in the hair of children aged 7–16 years [38]. However, we did not find any significant correlation between blood Cd and ADHD behaviors in Guiyu children. The lack of correlation is also seen in other surveys [39,40]. The possible explanation might be the relatively low level of blood Cd (0.95 ug/L) seen in our study comparing with 5 ug/L reported as the risk of intoxication [21]. Nevertheless, we found that BCLs were positively correlated with BLLs. Such an association might have compounding effects (e.g. agonistic) on neurotoxicity of children, which needs to be explored further [41,42].

It should be noted that our data are based solely on the Parent Rating Scale of the DSM-IV criteria of ADHD. We suspected that the actual prevalence of ADHD in preschool children in Guiyu might be lower than 12.8%, as the relatively incomprehensive evaluation procedures were used. A complete and standard procedure should contain ratings from both Parent and Teacher Rating Scale and ensure that the ADHD symptoms are not associated with other mental disorders such as pervasive developmental disorders. But the goal of our study was not aimed for diagnostic purposes, but rather to estimate the prevalence of ADHD symptomatology in preschool children**.** Moreover, it appears that the local physicians rely on Parent Rating Scale to diagnose ADHD, because there are a large number of children in this area who do not attend kindergarten, which makes it difficult to establish the comprehensive evaluation package without the Teacher Rating Scale. Another drawback is the lack of control group to confirm the association between e-waste exposure and ADHD. However, our previous study [9] and this study showed that the blood Pb might reflect the severity of e-waste exposure in Guiyu and point to the influence of e-waste exposure on childhood ADHD. Finally, a larger study population and other e-waste toxicants such as mercury and PAHs are needed in the future to evaluate risks of ADHD associated with the e-waste exposure.

## Conclusions

To our knowledge, very few studies have been conducted in ADHD of children living in primitive e-waste recycling areas. We analyzed the prevalence rate of the ADHD based on the DSM-IV Parent Rating Scale and its three subtypes (inattensive, hyperactive/impulsive and combined types) in Guiyu, China. We showed that children with high BLLs had 2.4 times higher risk of ADHD than those with low BLLs. The study may have policy implications for the local government to regulate e-waste recycling such that children’s access is limited and that recycling methods are to be improved to reduce exposure [43]. Furthermore, not only for Guiyu but for other developing countries where similar e-waste exposure scenarios exist, more attention should be paid for the environmental hazard such as the one we showed in this study.

## Additional file

Below is the link to the electronic supplementary material.

Additional file 1: **This file provides two questionnaires.** One is the risk assessment for childhood ADHD under e-waste exposure and the other is the Parent Rating Scale of DSM-IV ADHD criteria.

## Abbreviations

ADHD: Attention-deficit/hyperactivity disorder
E-waste: Electronic waste
BLLs: Blood lead levels
BCLs: Blood cadmium levels
DSM-IV: Diagnostic and statistical manual of mental disorders, 4th Edition

## References

[1] Puckett J, Byster L, Westervelt S, Gutierrez R, Davis S, Hussain A, Dutta M: Exporting Harm: The High-Tech Trashing of Asia. 2002. Available at: http://www.ban.org/E-waste/technotrashfinalcomp.pdf (accessed May 12^th^, 2014).

[2] Schmidt CW (2002). e-Junk explosion. Environ Health Perspect.

[3] Huo X, Peng L, Xu X, Zheng L, Qiu B, Qi Z (2007). Elevated blood lead levels of children in Guiyu, an electronic waste recycling town in China. Environ Health Perspect.

[4] Wong CS, Wu SC, Duzgoren-Aydin NS, Aydin A, Wong MH (2007). Trace metal contamination of sediments in an e-waste processing village in China. Environ Pollut.

[5] Yu XZ, Gao Y, Wu SC, Zhang HB, Cheung KC, Wong MH (2006). Distribution of polycyclic aromatic hydrocarbons in soils at Guiyu area of China, affected by recycling of electronic waste using primitive technologies. Chemosphere.

[6] Leung AO, Luksemburg WJ, Wong AS, Wong MH (2007). Spatial distribution of polybrominated diphenyl ethers and polychlorinated dibenzopdioxins and dibenzofurans in soil and combusted residue at Guiyu, an electronic waste recycling site in southeast China. Environ Sci Technol.

[7] Brigden K, Labunska I, Santillo D, Allsopp M: Recycling of electronic wastes in China and India: workplace and environmental contamination. 2005. Available at: http://www.greenpeace.org/india/press/reports/recycling-of-electronic-wastes (accessed April 5^th^, 2014).

[8] Qiu B, Peng L, Xu X, Lin X, Hong J, Huo X (2004). Medical Investigation of E-waste de Manufacturing Industry in Guiyu Town. Proceedings of the International Conference on Electronic Waste and Extended Producer Responsibility: 21–22 April 2004.

[9] Zheng L, Wu K, Li Y, Qi Z, Han D, Zhang B (2008). Blood lead and cadmium levels and relevant factors among children from an e-waste recycling town in China. Environ Res.

[10] Barkley RA (1990). Attention-deficit Hyperactivity Disorder: A Handbook for Diagnosis and Treatment.

[11] American Academy of Pediatrics (2000). Clinical practice guideline: diagnosis and evaluation of the child with attention-deficit/hyperactivity disorder. Pediatrics.

[12] Erhart M, Herpertz-Dahlmann B, Wille N, Sawitzky-Rose B, Höllin H, Ravens-Sieberer U (2012). Examining the relationship between attention-deficit/hyperactivity disorder and overweight in children and adolescents. Eur Child Adolesc Psychiatry.

[13] Hanć T, Słopień A, Wolańczyk T, Dmitrzak-Węglarz M, Szwed A, Czapla Z, Durda M, Ratajczak J, Cieślik J: ADHD and overweight in boys: cross- sectional study with birth weight as a controlled factor. Eur Child Adolesc Psychiatry 2014, in press. 10.1007/s00787-014-0531-1 PMC4291509 24633695

[14] Rowland AS, Umbach DM, Catoe KE, Stallone L, Long S, Rabiner D (2001). Studying the epidemiology of attention- deficit hyperactivity disorder: screening method and pilot results. Can J Psychiatry.

[15] Froehlich TE, Anixt JS, Loe IM, Chirdkiatgumchai V, Kuan L, Gilman RC (2011). Update on environmental risk factors for attention-deficit/hyperactivity disorder. Curr Psychiatry Rep.

[16] Goodlad JK, Marcus DK, Fulton JJ (2013). Lead and attention-deficit/hyperactivity disorder (ADHD) symptoms: A meta-analysis. Clin Psychol Rev.

[17] Ciesielski T, Weuve J, Bellinger DC (2012). Cadmium exposure and neuro- developmental outcomes in US children. Environ Health Perspect.

[18] Cheong H, Kwon H, Kim E, Ha M (2008). Blood manganese level and attention deficit/hyperactivity disorder in early school age children. Epidemiolog.

[19] Polańska K, Jurewicz J, Hanke W (2012). Exposure to environmental and lifestyle factors and attention-deficit/hyperactivity disorder in children—a review of epidemiological studies. Int J Occup Med Environ Health.

[20] Centers for Disease Control: Preventing in lead poisoning in young children. 1991. Available at: http://wonder.cdc.gov/wonder/prevguid/p0000029/p0000029.asp#head0010000000000001991,1-74 (accessed January 10^th^, 2012).

[21] Jin T, Nordberg M, Frech W, Dumont X, Bernard A, Ye TT (2002). Cadmium biomonitoring and renal dysfunction among a population environmentally exposed to cadmium from smelting in China (ChinaCad). Biometals.

[22] American Psychiatric Association (1994). Origin of Diagnostic and Statistical Manual of Mental Disorders.

[23] Skounti M, Philalithis A, Galanakis E (2007). Variations in prevalence of attention deficit hyperactivity disorder worldwide. Eur J Pediatr.

[24] Gimpel GA, Kuhn BR (2000). Maternal report of attention deficit hyperactivity disorder symptoms in preschool children. Child Care Health Dev.

[25] Magnússon P, Smári J, Grétarsdóttir H, Prándardóttir H (1999). Attention-deficit/hyperactivity symptoms in Icelandic schoolchildren: assessment with the attention deficit/hyperactivity rating scale-IV. Scand J Psychol.

[26] Skounti M, Philalithis A, Mpitzaraki K, Vamvoukas M, Galanakis E (2006). Attention deficit hyperactivity disorder in schoolchildren in Crete. Acta Paed.

[27] Roy A, Bellinger D, Hu H, Schwartz J, Ettinger AS, Wright RO (2009). Lead exposure and behavior among young children in Chennai, India. Environ Health Perspect.

[28] Nigg JT, Nikolas M, Mark Knottnerus G, Cavanagh K, Friderici K (2010). Confirmation and extension of association of blood lead with attention-deficit/hyperactivity disorder (ADHD) and ADHD symptom domains at population-typical exposure levels. J Child Psychol Psychiatry.

[29] Wang JP, Guo XK (2006). Impact of electronic wastes recycling on environmental quality. Biomed Environ.

[30] Cantwell DP (1996). Attention deficit disorder: a review of the past 10 years. J Am Acad Child Adolesc Psychiatry.

[31] Biederman J, Mick E, Faraone SV (2000). Age-dependent decline of symptoms of attention deficit hyperactivity disorder: impact of remission definition and symptom type. Am J Psychiatry.

[32] Konofal E, Lecendreux M, Arnulf I, Mouren MC (2004). Iron deficiency in children with attention-deficit/hyperactivity disorder. Arch Pediatr Adolesc Med.

[33] Linver MR, Brooks-Gunn J, Kohen DE (2002). Family processes as pathways from income to young children’s development. Dev Psychol.

[34] Max W, Sung HY, Shi Y (2013). Attention deficit hyperactivity disorder among children exposed to secondhand smoke: a logistic regression analysis of secondary data. Int J Nurs Stud.

[35] Froehlich TE, Lanphear BP, Auinger P, Hornung R, Epstein JN, Braun J (2009). Association of tobacco and lead exposures with attention-deficit/hyperactivity disorder. Pediatrics.

[36] Capel ID, Pinnock MH, Dorrell HM, Williams DC, Grant EC (1981). Comparison of concentrations of some trace, bulk, and toxic metals in the hair of normal and dyslexic children. Clin Chem.

[37] Thatcher RW, Lester ML, McAlaster R, Horst R (1982). Effects of low levels of cadmium and lead on cognitive functioning in children. Arch Environ Health.

[38] Bao QS, Lu CY, Song H, Wang M, Ling W, Chen WQ (2009). Behavioural development of school-aged children who live around a multi-metal sulphide mine in Guangdong province. China: a cross-sectional study. BMC Public Health.

[39] Szkup-Jabłońska M, Karakiewicz B, Grochans E (2012). Effects of blood lead and cadmium levels on the functioning of children with behaviour disorders in the family environment. Ann Agric Environ Med.

[40] Kim S, Arora M, Fernandez C, Landero J, Caruso J, Chen A (2013). Lead, mercury, and cadmium exposure and attention deficit hyperactivity disorder in children. Environ Res.

[41] Antonio MT, Corpas I, Leret ML (1999). Neurochemical changes in newborn rat’s brain after gestational cadmium and lead exposure. Toxicol Lett.

[42] de Burbure C, Buchet JP, Leroyer A, Nisse C, Haguenoer JM, Mutti A (2006). Renal and neurologic effects of cadmium, lead, mercury, and arsenic in children: evidence of early effects and multiple interactions at environmental exposure levels. Environ Health Perspect.

[43] Trasande L, Massey RI, DiGangi J, Geiser K, Olanipekun AI, Gallagher L (2011). How developing nations can protect children from hazardous chemical exposures while sustaining economic growth. Health Aff (Millwood).

