# Supplementary material for: Attention-deficit/hyperactivity symptoms in preschool children from an E-waste recycling town: assessment by the parent report derived from DSM-IV
Source: BMC Pediatr. 2015 May 5;15:51. doi: 10.1186/s12887-015-0368-x (PMC4429982; doi:10.1186/s12887-015-0368-x)
Supplement: Supplementary file 1 — This file provides two questionnaires. One is the risk assessment for childhood ADHD under e-waste exposure and the other is the Parent Rating Scale of DSM-IV ADHD criteria. [file 12887_2015_368_MOESM1_ESM.doc]

**Attention-Deficit/Hyperactivity Symptoms** **in Preschool Children from** **an E-waste Recycling Town: Assessment by** **the Parent Report Derived from DSM-IV**

Authors: Ruibiao Zhang, Xia Huo, Guyu Ho, Xiaojuan Chen, Hongwu Wang, Tianyou Wang, Lian Ma.

**Additional file 1**

**Questionnaire 1: Risk assessment for childhood ADHD under e-waste exposure**

Name: Gender: D.O.B. :

Home address: Phone number:

Completed by: Mother____Father____Other____Date of completion:

| 1. Your child’s birth weight was_____kg. |  |
| --- | --- |
| 2. Your child’s gestational age was_____weeks. |  |
| 3. Has your child ever experienced a head injury, loss of  consciousness, or seizure?  If yes, please specify ______________. | □Yes □No |
| 4. Does your child have any developmental delays in  language or movement? | □Yes □No |
| 5. Has your child ever been diagnosed as ADHD? | □Yes □No |
| 6. Is your child currently taking medications?  If yes, please specify ______________. | □Yes □No |
| 7. How often does your child take milk products? | □Daily □Weekly  □Monthly □Less than monthly |
| 8. How often does your child take soybean products? | □Daily □Weekly  □Monthly □Less than monthly |
| 9. Does your child have hand-to-mouth behavior, or put  non-food items such as paint chips or soil, in his/her mouth? | □Yes □No |
| 10. How many hours does your child play outside near the  road everyday? | □Within half an hour □One hour  □Two hours□More than two hours |
| 11. Does your child live in a house that is or was serving as  an e-waste workshop? | □Yes □No |
| 12. Does your child live in a house that was renovated  within the last 12 months or is currently being renovated? | □Yes □No |
| 13. How many e-waste workshops around your house  within 50 meters? | □None □1-2  □3-4 □5 or more |
| 14. How often does anyone smoke inside your house? | □Daily □Weekly  □Monthly □Less than monthly |
| 15. What is the highest level of education the child’s  father completed? | □Illiterate/primary school  □Middle school  □High school  □College |
| 16. Does the father engage in the type of work related  to e-waste?  If yes, please give the detail type_____________. | □Yes □No |
| 17. What is the highest level of education the child’s  mother completed? | □Illiterate/primary school  □Middle school  □High school  □College |
| 18. Does the mother engage in the type of work related  to e-waste?  If yes, please give the detail type_______________. | □Yes □No |
| 19. What is your total monthly household income?  (Including all sources – salary, bonuses, investment income  and so on) | □Less than 2000 yuan  □2000-5000 yuan  □More than 5000 yuan |

**Questionnaire 2: The Parent Rating Scale of DSM-IV ADHD criteria**

Child’s name: Age: Gender:

Completed by: Date:

Check the box that describes the child's behavior at home over the past six months.

| Items | Never  or rarely | Sometimes | Often | Very  often |
| --- | --- | --- | --- | --- |
| **Inattention subscale** |  |  |  |  |
| 1. Fails to give close attention to details or makes careless  mistakes in school work, work, or other activities |  |  |  |  |
| 2. Has difficulty sustaining attention in tasks or playing |  |  |  |  |
| 3. Does not seem to listen when spoken to directly |  |  |  |  |
| 4. Does not follow through on instructions and fails to finish  schoolwork, chores, or duties in the workplace (not due to  oppositional behavior or failure to understand instructions) |  |  |  |  |
| 5. Has difficulty organizing tasks and activities |  |  |  |  |
| 6. Avoids, dislikes, or is reluctant to engage in tasks that require  sustained mental effort (e.g. schoolwork or homework) |  |  |  |  |
| 7. Loses things necessary for tasks or activities (e.g., toys, school  assignments, pencils, books, or tools) |  |  |  |  |
| 8. Is easily distracted by extraneous stimuli |  |  |  |  |
| 9. Is forgetful in daily activities |  |  |  |  |
| **Hyperactivity/Impulsivity subscale** |  |  |  |  |
| 10. Fidgets with hands or feet or squirms in seat |  |  |  |  |
| 11. Leaves seat in classroom or in situations in which remaining  seated is expected |  |  |  |  |
| 12. Runs about or climbs excessively in situations in which it is  inappropriate (in adolescents may be feelings of restlessness) |  |  |  |  |
| 13. Has difficulty playing or engaging in leisure activities quietly |  |  |  |  |
| 14. Is “on the go” or often acts as if “driven by a motor” |  |  |  |  |
| 15. Talks excessively |  |  |  |  |
| 16. Blurts out answers before questions have been completed |  |  |  |  |
| 17. Has difficulty awaiting turn |  |  |  |  |
| 18. Interrupts or intrudes on others (e.g. butts into conversations  or games) |  |  |  |  |
